# Conidial Emulsion Formulation and Thermal Storability of *Metarhizium anisopliae* against Red Palm Weevil, *Rhynchophorus* *ferrugineus* Olivier (Coleoptera: Dryophthoridae)

**DOI:** 10.3390/microorganisms10071460

**Published:** 2022-07-19

**Authors:** Cheong Jia Lei, Najihah Abdul Halim, Norhayu Asib, Azlina Zakaria, Wahizatul Afzan Azmi

**Affiliations:** 1 Faculty of Science and Marine Environment, Universiti Malaysia Terengganu, Kuala Nerus 21030, Terengganu, Malaysia; cheongjialei@gmail.com (C.J.L.); najihahhalim_94@yahoo.com (N.A.H.); 2 Department of Plant Protection, Faculty of Agriculture, Universiti Putra Malaysia, Serdang 43400, Selangor, Malaysia; norhayuasib@upm.edu.my; 3 Sime Darby Research Sdn. Bhd., KM10, Jalan Banting-Kelanang, P.O. Box 207, Banting 42700, Selangor, Malaysia; azlina.zakaria@simedarbyplantation.com

**Keywords:** *Metarhizium anisopliae*, entomopathogenic fungus, green muscardine disease, *Rhynchophorus ferrugineus*, red palm weevil, nanoemulsion formulation, oil-in-glycerol emulsion, mycoinsecticide, conidia, Met-Gra 4

## Abstract

Industrial crops including coconut palm and other palm species are seriously infested by red palm weevil (RPW), resulting in significant economic damage globally. Therefore, this study aimed to develop a mycoinsecticide utilizing conidia of *Metarhizium anisopliae* to control RPW and sought to investigate a new emulsion formulation for the influences of storage temperature and heat stress on conidia germination in an oil-in-glycerol emulsion system. The mycoinsecticide is an emulsion formulation which comprises an oil carrier, non-ionic surfactants, water, and glycerol, which was optimized by premixing the oil and non-ionic surfactant in different weight ratios (1:0, 9:1, 8:2, 7:3, 6:4, 5:5, 4: 6, 3: 7, 2:8, 1:9, and 0:1). From three selected oil-in-glycerol formulations, F25 was more stable in storage and had a smaller particle size (between 154.3 and 236.4 nm in diameter) and stable zeta potential (above + 30 mV) with low surface tension (29.83 ± 0.24 mN/m to 30.72 ± 0.11 mN/m at room temperature. Extended conidial viability was observed at 4 °C overall; the emulsion formulation maintained 12–15% conidial viability until the eighth week at room temperature. Heat of over 30 °C showed an inhibitory effect on conidial germination. This study revealed that the oil-in-glycerol formulation was stable and able to prolong conidial shelf life as compared to non-formulated conidia.

## 1. Introduction

The control of the red palm weevil (*Rhynchophorus ferrugineus*) in Malaysia is broadly based on the use of synthetic insecticides, with some commercially available products, such as cypermethrin and dimethoate [1,2]. The non-selective use of these products, either way, may select for weevil populations resistant to chemical insecticides, lead to severe chemical pollution to the environment, and may increase risks to non-target organisms and human health [3]. The biological control of RPW with entomopathogenic fungi (EPF) has been suggested as a promising alternative method to the exclusive use of chemicals [4]. Malaysia has several marketable biological control agents and mostly exploits fungal products using *Metarhizium* spp. as the key active ingredient. However, no fungal products are registered for RPW control in Malaysia.

Abiotic factors may restrain the development of *Metarhizium* spp. and lower the efficacy in biological control [5]. The pathogenicity against the insect host and the field persistence of fungal products are directly influenced by high temperature [6] or low relative humidity [7]. Thus, formulations could be essential to increase fungal tolerance to ecophysiological factors and enhance the biological performance of EPF against RPW. Moreover, an appropriate formulation is also crucial to carry and disperse fungal propagules while prolonging their viability. As an initiative in this study, vegetable oils served as a carrier and non-ionic surfactants were mixed at a proper ratio to oil and water, aiming to develop a new, effective, and economic conidial formulation in an oil emulsion. 

The production of a large number of conidia is crucial for mycoinsecticide commercialization, yet the success of fungal-based bioinsecticides also depends on storage conditions. Additionally, environmental stresses in the field (such as high temperature) may also cause a deleterious effect on the EPF’s effectiveness by reducing the pathogenicity and conidial viability [8]. An oil emulsion formulation which generally comprises an oil carrier, surfactant(s), and water has been shown to enhance the conidial shelf life and resistance to adverse environmental stresses throughout field application, which is critical for the efficacy of mycoinsecticides [9].

In comparison with the non-formulated conidia, oil emulsion formulation prolongs the shelf life and improves the pathogenicity of entomopathogenic fungi against the insect pests, such as the citrus psyllids [10] and mealybugs [11]. Various oil carriers, namely mineral and sunflower oil, have been used to prepare oil-based formulations [12,13]. Additionally, sesame oil was also found to prolong the food shelf life owing to the high content of polyunsaturated fats [14], suggesting the potential of oils as an effective carrier for the formulation of bioinsecticides. 

The previous study by Grace et al. [15] showed the efficacy of *M. anisopliae* strain Met-Gra4 against RPW. However, there has been no further investigation about the effect of different emulsion components on storage stability and fungal thermal tolerance. Therefore, the objectives of this study were: (a) to examine the effect of commercially available vegetable oils, non-ionic surfactants, and other inert ingredients on conidial viability, (b) to assess the emulsion stability in different combinations of inert ingredients, (c) to attempt in reducing non-ionic surfactant content for a stable emulsion system while considering cost-effectiveness, (d) to evaluate the shelf life of both the emulsion-formulated conidia and non-formulated conidia at two storage conditions (4 °C and 28 °C), and (e) to examine the thermal tolerance of both the emulsion-formulated conidia and non-formulated conidia with a simulation of room temperature (25 °C) to the extreme temperatures in the field (up to 45 °C).

## 2. Materials and Methods

### 2.1. Materials

Pure culture of *M. anisopliae* strain Met-Gra4 was obtained from Grace Lee (PhD., UMT), as nominated in her publication [15]. The conidia of Met-Gra4 were grown and mass-produced by solid substrate fermentation, then dry-harvested through a sieve shaker. The drying temperatures for post-fermentation solid substrate were maintained at room temperature (25 ± 3 °C). The quality of harvested conidia was analyzed before formulation preparation. 

There were three categories of ingredients, namely vegetable oils and derivatives, co-stabilizers, and non-ionic surfactants in the study. The Department of Plant Protection (UPM) provided non-ionic surfactants (Agnique^®^ PG9116, Agnique^®^ PG8105, Termul^®^ 1284, Tween^®^ 20, Tween^®^ 80, Triton^TM^ X-100, Span^®^ 20, Span^®^ 80, and Span^®^ 85), vegetable oils (canola oil, castor oil, palm oil, sesame oil, sunflower oil, and virgin coconut oil), and oil derivatives (palm kernel oil ester and rapeseed methyl ester). Capric oil was purchased from Take It Global Sdn. Bhd. (Malaysia). Glycerol was purchased from Sigma-Aldrich. Moreover, sodium alginate, gum Arabic, inulin powder, tapioca starch, and wheat starch were used as co-stabilizers as suggested by Yang et al. [16]. Deionized water was utilized for preparing all mixtures and emulsions. All concentrations were standardized to the mass percentage (% *w*/*w*). 

### 2.2. Active and Inert Ingredient Compatibility Screening

The compatibility of dry-harvested conidia of *M. anisopliae* (95 ± 3% viable conidia; strain Met-Gra4) was tested against several inert ingredients, wherein 0.01 g of dried conidia was suspended either in 1 mL of oils, an aqueous mixture of co-stabilizers, or non-ionic surfactants (10%, 20%, or 30% aqueous solution) and incubated for 24 h at room temperature for three replicates. To ensure an appropriate amount of colony-forming units (CFUs) for plate counting (30–300 conidia), 30 μL of each conidial suspension (of all tested ingredients) was then inoculated on potato dextrose agar (PDA) upon dark incubation at 28 °C. The conidial viability percentage of each agar plate was determined after 24–36 h of incubation. The ingredients which maintained at least 70% conidial viability were considered suitable for the subsequent steps. 

### 2.3. Emulsion Formulation Preparation

Emulsion formation was performed by admixing compatible oils and non-ionic surfactants with the least fungal growth inhibitory effect. The method was based on spontaneous or vortex-assisted emulsification as described by Chouhan & Saini [17]. In brief, surfactants were either used alone or blended according to the required hydrophile–lipophile balance (HLB) value for oils. Emulsification was performed by premixing oils and surfactants (or surfactant blend) in different weight ratios (1:0, 9:1, 8:2, 7:3, 6:4, 5:5, 4:6, 3:7, 2:8, 1:9, and 0:1) into different glass vials to a total of 0.5 g. The prepared compositions were then centrifuged for three minutes at 3000 rpm and vortexed for one minute to obtain a homogenous mixture. A total of 10% *w*/*w* of water or glycerol was titrated drop-wise to the oil–surfactant mixture until a water or glycerol content of 90% was achieved in the emulsion system. The mixture was then vortexed and centrifuged (3000 rpm; 10 min) after each titration. The resultant mixture was visually examined to determine their phase behavior and transition based on transparency. The phase behavior of the mixture was categorized as isotropic (p_i_; one phase; transparent), homogenous (H; one phase; translucent or opaque; cloudy or milky), or anisotropic (p_ii_; multiple phase separations). Pseudo-ternary phase diagrams were mapped using CHEMIX School version 8.0 software. 

### 2.4. Incorporation of Conidia in Emulsion System

The formulation was selected from the pseudo-ternary phase diagram, prioritizing being optically isotropic, transparent, one-phase, and thermodynamically stable at room temperature and its surfactant concentration being equal to and below 20%. Different Met-Gra4 dry conidia (% *w*/*w*) and oil/glycerol (% *w*/*w*) ratios were used for developing a stable emulsion system. The conidia contents were varied (5, 10, 15, 20, 25, and 30% *w*/*w*) at different oil/glycerol ratios, selected from the isotropic region of the pseudo-ternary phase diagrams. A high-pressure homogenizer was used carefully to mix conidia at a maximum of 20,000 rpm. Emulsion stability was monitored by centrifuging the resultant mixture at 3000 rpm for 15 min. Emulsions were then poured into flat-bottomed cylindrical glass tubes and stored at 25 ± 1 °C for one week. Conidial viability was examined in emulsion stock and diluted formulation (50% *w*/*w*). Efficient and stable conidial emulsion formulations which could maintain the ungerminated conidia before dilution were selected.

### 2.5. Characterization of Emulsion Formulations

The selected formulation was prepared and kept at a low temperature (4 ± 1 °C), room temperature (25 ± 1 °C), and high temperature (54 ± 1 °C) for 90 days to assess the phase behavior and thermostability of the pre-formulation, as suggested by Lim et al. (2012) in the nanoformulation mechanism study. The physical appearance of the formulations was visually examined. The particle size distribution and zeta potential analysis were conducted using Zetasizer Nano ZS (Malvern Instruments, Malvern, UK). This instrument detects the micellar size from intensity–time fluctuations of a ‘red’ laser beam (4 mW or 10 mW; 632.8 nm; 175°). 

Each measurement was run an average of three times to prevent the sample from overheating. To avoid multiple scattering effects, formulations were diluted at a ratio of 50 µL to 100 mL of deionized water before the particle size measurements. Result values corresponding to the mean ± standard deviation (SD) of three measurements of each formulation were reported. The instrument which calculated the polydispersion index (PDI) reflects the homogeneity profile of the droplet diameter and measured for formulation stored on the 0, 30th, and 60th day at both temperature settings. A rotary viscometer was used to measure the dynamic viscosities of all formulations at 25 ± 1 °C. The pH value of the emulsions was determined using a desktop pH meter at room temperature (25 ± 1 °C). Lastly, a survismeter was used to determine surface tension for the tested formulations.

### 2.6. Effect of Storage Temperature on Conidial Viability

The emulsion-formulated conidia and non-formulated control (dry-harvested conidia in 0.05% aqueous Tween 80 suspension) were examined at 4 °C and 28 °C for eight weeks. The emulsion of formulated and non-formulated Met-Gra4 conidia viability was assessed by taking 0.5 g samples at weekly intervals. Each plate was inoculated with 20 μL conidia suspension, at 10^6^–10^7^ conidia mL^−1^, evenly spread with a glass rod on the surface of PDA supplemented with 0.02% chloramphenicol (g/L), then incubated for 18 h at 28 °C. Dry-harvested conidia were added to 0.02% aqueous solution before inoculation. 

Conidia were scored for the presence of germ tubes under a compound microscope (400× magnification). Conidia were considered germinated if the germ tube length was longer than the length of the conidium. Roughly around 200 conidia were counted on each plate. The average percent conidial viability of three replicates was determined at each sampling time. Conidial viability was assessed repeatedly for both the emulsion-formulated and non-formulated conidia stored for 12 weeks, at 4 °C and 28 °C, according to Alves et al. (2002) [6], with some modifications on temperature settings. Both formulated and non-formulated conidia emulsions were examined for premature germination before incubation, avoiding a false-positive result.

### 2.7. Effect of Heat Stress on Conidial Viability

Conidial viability for all formulations was examined based on de Oliveira et al. [18] with slight modifications: (a) the one-hour interval of observation for conidial viability was adjusted to the four-hour interval, and (b) each temperature setting was adjusted in 5 °C increments until 45 °C. In brief, formulated and non-formulated conidia samples were mixed directly in 0.05% aqueous Tween 80 and vortexed. The suspension tube was centrifuged for five minutes at 12,000 rpm at 4 °C. The supernatant was discarded, and 0.1 g of the conidia precipitate was carefully removed for further dilution. 

Conidial suspensions for every formulation were standardized to a final concentration of 1 × 10^6^ conidia mL^−1^. Suspension replicates of both formulated and non-formulated conidia were prepared in 0.05% aqueous Tween 80 at 25 °C. The suspensions were pipetted into 2.0 mL microcentrifuge tubes and kept at 25 °C, 30 °C, 35 °C, 40 °C, and 45 °C in the dark. The temperature of 25 °C served as control (standard room temperature), while temperatures above 35 °C were used to simulate extreme temperatures faced by farmers in the field. 

Both formulated and non-formulated conidia were examined for premature germination before incubation, avoiding a false-positive result at 0 h. Conidial viability was scored after 4, 8, and 12 hours under thermal incubation stress. Petri dishes containing 6 mL of PDA, supplemented with 0.02% chloramphenicol (g/L), were inoculated with 50 µL of conidial suspension. The plates were sealed after evaporating excess liquid, then incubated at 28 °C. Plate counting of the viable and non-viable conidia was evaluated under a compound microscope at 400× magnification. Viable conidia were identified by their protruding germ tubes being longer than their diameters.

### 2.8. Statistical Analyses

The mean value of three replicates for each treatment was used throughout the statistical analyses. Standard deviation values were calculated for each treatment. Analyses were conducted using IBM SPSS Statistics 25 software. A certain treatment dataset was checked for normality. When the data were normally distributed, univariate analysis using two-way ANOVA with Tukey’s honestly significant difference (HSD) test was conducted. Univariate analysis was conducted on various surfactant types and concentrations on conidial viability.

Furthermore, when the data were normally distributed, three-way ANOVA with Tukey’s honestly significant difference (HSD) test was carried out to determine significant differences in the conidial viability in emulsion formulation between the different treatments. For conidia viability under heat stress, Kruskal–Wallis H test with post hoc pairwise comparison was conducted to determine the significant differences in heat stress treatments.

## 3. Results

### 3.1. Inert Ingredient Screening

**Figure 1:**
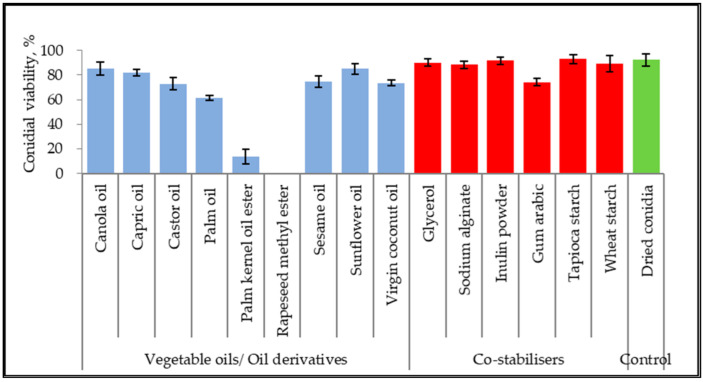
Conidial viability of *M. anisopliae* (strain Met-Gra4) suspended in 100% oils and aqueous mixture of co-stabilizers. Data are expressed as mean ± standard deviation (*n* = 3).

The germination inhibitory effect of seven vegetable oils, two vegetable oil ester derivatives, ten non-ionic surfactants, and six co-stabilizers were assessed with dry-harvested conidia of *M. anisopliae,* as shown in Figure 1. The conidial viability was examined based on germ tube formation. Untreated control of dried conidia achieved 92.33 ± 4.93% viability. The viability of conidia suspended in canola oil, capric oil, and sunflower oil reached 85.33 ± 5.03%, 82.00 ± 2.65%, and 85.00 ± 4.36%, respectively. Both oil ester derivatives inhibited conidial germination at 0–8%. Additionally, other unmentioned vegetable oils scored between 61.67% to 73.67%. All the co-stabilizers showed the least or no inhibitory effect on conidial viability. 

**Figure 2:**
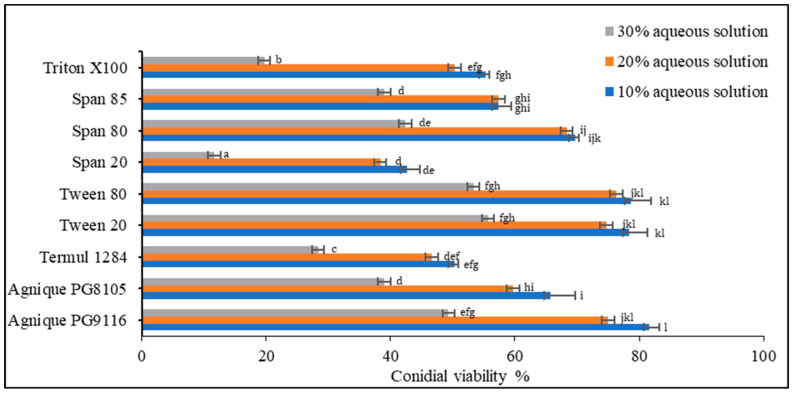
Conidial viability of *M. anisopliae* (Met-Gra4) conidia with different concentrations of aqueous surfactant solution. Data expressed as mean ± standard deviation (*n* = 3). According to Tukey’s HSD test, the means of conidial viability (%) of each surfactant and between tested concentrations followed by different letters indicate significant differences at *p* = 0.05.

Based on the screening of nine non-ionic surfactants on dry-harvested conidia (Figure 2), Levene’s test of equality of error variances indicated sufficient evidence to claim that the variances were equal across groups (*F*_0.05,26,54_ = 2.529, *p* = 0.02) Data showed a significant difference among the three concentrations (*p* < 0.005). Negative control using deionized water showed no inhibitory effect on conidial germination. Two-way analysis of variance (ANOVA) demonstrated significant differences among surfactants (*p* < 0.005), except Agnique PG9116, Tween 20, and Tween 80. At the concentration of 30% of the surfactant, conidial viability in all treatments plummeted below 55%, unsuitable for emulsion formulation preparation. Only Agnique PG9116, Tween 20, and Tween 80 met the selection criteria, maintaining 74.67–81.67% of viable conidia, while Span 80 (68.33–69.67%) was selected as the co-surfactant to adjust the HLB value according to the required HLB of oils.

### 3.2. Phase Behaviour Study of the Mixtures 

Based on the previous screening of the suitable oil, five vegetable oils that maintained at least 70% of viable conidia were chosen and used to prepare the emulsion system, as listed in Table 1. A single surfactant showed no ability to create a stable emulsion system where flocculation and sedimentation were observed, except castor oil/Tween 80 or Tween 20/water system. Thus, the following combination of vegetable oils and surfactant blends was examined according to the required HLB value (Table 1).

**Figure 3:**
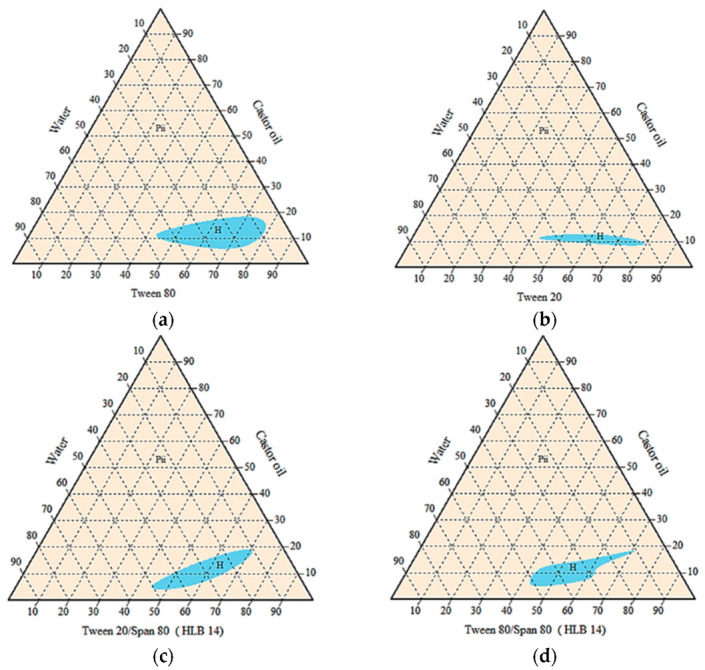
Pseudo-ternary phase diagrams of castor oil/surfactant/water systems without active ingredients, using single or mixed surfactants (**a**) Tween 80, (**b**) Tween 20, (**c**) Tween 20:Span 80, and (**d**) Tween 80:Span 80. H = homogenous cloudy or milky; P_ii_ = multiphase.

Agnique PG9116 was excluded due to its inability to form a homogenous emulsion with any vegetable oils blended. Pseudo-ternary phase diagrams below (Figure 3) showed that the castor oil/surfactant/water system with the largest isotropic region of cloudy mixture was achieved by Tween 80 alone. Oil-in-water emulsions were formed as indicated by less than 20% of castor oil blended. However, the amount of surfactant needed was considerably high, between 45% to 80% for all homogenous systems. 

**Figure 4:**
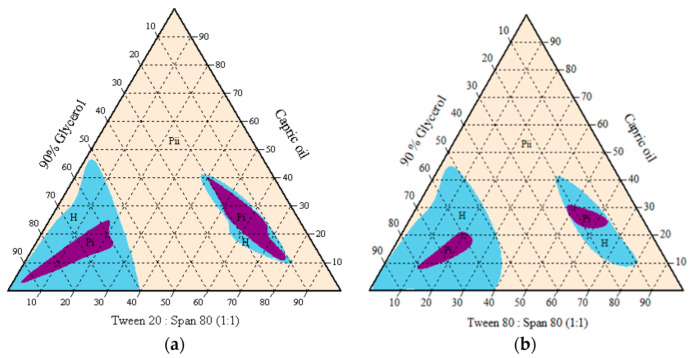
Pseudo-ternary phase diagrams of capric oil/surfactant/water/glycerol systems without active ingredients, using single or mixed surfactants (**a**) Tween 80, and (**b**) Tween 20. H = homogenous cloudy and milky; P_i_ = one-phase/ transparent; P_ii_ = multiphase.

The subsequent emulsion system was then modified with the aqueous phase changed to 90% glycerol due to premature germination in the oil-in-water emulsion system. Only medium-chain triglyceride (capric oil) and surfactant blend were compatible in forming the isotropic homogenous one-phase region. An oil-in-glycerol emulsion with watery to gluey consistency was observed at less than 40% surfactant blended, while a higher amount of surfactant generally formed a gel-like mixture (Figure 4). The isotropic one-phase region indicated a smaller particle size in a transparent or translucent resultant mixture. The surfactant blend of Tween 20 and Span 80 covered a larger one-phase region than the surfactant blend of Tween 80 and Span 80. The transparent mixtures appeared stable at room temperature (25 °C) for more than six months. 

**Figure 5:**
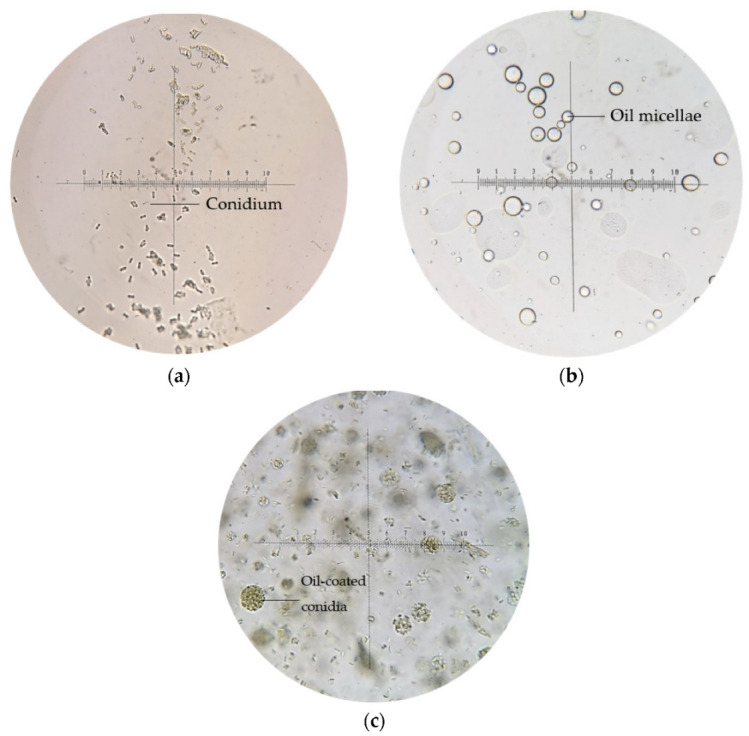
Microscopic images of (**a**) conidia suspension in water, (**b**) emulsion blank, and (**c**) emulsion-formulated conidia, at 400× magnification.

A total of 50 points was selected from the one-phase/homogenous cloudy region before adding 0.1–10% dried conidia (in the weight of formulation blank) to observe any emulsion instability or phase separation. Dry-harvested conidia were blended only with emulsion systems that contained less than 20% surfactant. Due to the lipophilic properties of Met-Gra4 conidia, phase separation and sedimentation occurred rapidly in all types of oil-in-water and water-in-oil emulsions, regardless of the oil or surfactant used. However, stable oil-in-glycerol mixtures with 0.1–10% of the active ingredient were recorded at the ratio of 12:8:80, 15:10:75, and 20:9:71 (oil/Tween 20:Span 80/glycerol), known as F25, F31, F50, respectively. Phase separation occurred after two weeks for F31 and F50 at room temperature and three weeks in refrigerated conditions at 4 °C. F25 remained stable for more than one month (Figure 3, Figure 4 and Figure 5). The addition of gelatinized starch (co-stabilizer) caused separation within two days due to its larger and denser particle size. Gelatinized starch was observed to act as a growing medium causing conidia to germinate. At the same time, the addition of 0.1–10% of sodium alginate or inulin showed sedimentation in two days. Thus, those natural polymers were not used for the desired emulsion system as co-stabilizers. 

Generally, conidia suspended in water appeared singly or clumped, non-motile, and ellipsoidal (5–6 μm); multiple transparent oil micellae were visible in emulsion F25 formed by spontaneous emulsification as shown in Figure 5b, while the conidia-loaded emulsion led a pack of conidia coated within oil micellae, with excess conidia suspended freely within the emulsion body. 

**Figure 6:**
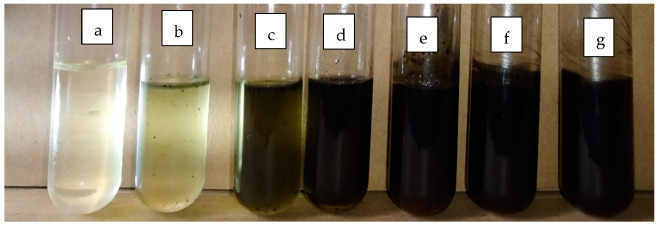
Image of emulsion F25 in the test tube varying in conidia content; (**a**) emulsion blank, emulsion loaded with conidia at (**b**) 0.1%, (**c**) 0.5%, (**d**) 1%, (**e**) 5%, (**f**) 7.5%, and (**g**) 10%.

The transparency of the emulsion blank and conidia formulated with emulsion F25 were shown in glass tubes containing 0 to 10% of conidia (Figure 6). For the subsequent assessment, the emulsion F25 was used throughout, thus any mention of emulsion formulation afterward refers to emulsion F25.

### 3.3. Characterization of Emulsion Formulations

The emulsion blank of F25 was thermostable with no phase separation at a low temperature (4 ± 1 °C), room temperature (25 ± 1 °C), and high temperature (54 ± 1 °C) within 90 days of storage. In contrast, a subsequent increase in temperature led to phase separation in the conidia-loaded emulsion. However, the viability of conidia at the separated phase, either in capric oil or 90% glycerol, had no significant difference from the homogenous emulsion. The conidia-loaded emulsion was able to regain homogenously through spontaneous shaking. The particle size distribution and zeta potential analysis of the undisturbed emulsion blank and the conidia-loaded emulsion were as presented in Table 2. 

Based on the zeta measurement, the emulsion blank was considered stable over 90 days and achieved nanometric particle sizes between 154.3 and 236.4 nm in diameter. The PDI value indicated that particles were uniformly dispersed, while a zeta potential above + 30 mV conferred stability at emulsion pH values of 5.2 to 7.4. Surface tension for the emulsion blank and emulsion loaded with conidia was similar, scored at 29.83 ± 0.24 mN/m and 30.72 ± 0.11 mN/m at room temperature, respectively.

On the other hand, the conidia-loaded emulsion showed a drastic increase in the Z-average from day 30 of storage and remained similar until day 90. The conidia-loaded emulsion sample was very polydisperse or contained large particles, aggregates, or sediments. An increasing count rate and size were observed, as the conidia may have been aggregating. A higher PDI value indicated multiple molecular weights, where the oil particles or conidia clumps were present. Attraction overcame the repulsion of particles as characterized by the low zeta potential, as the conidia-loaded emulsion was likely to form coagulates or sediments.

### 3.4. Conidial Viability at Storage Temperature

**Figure 7:**
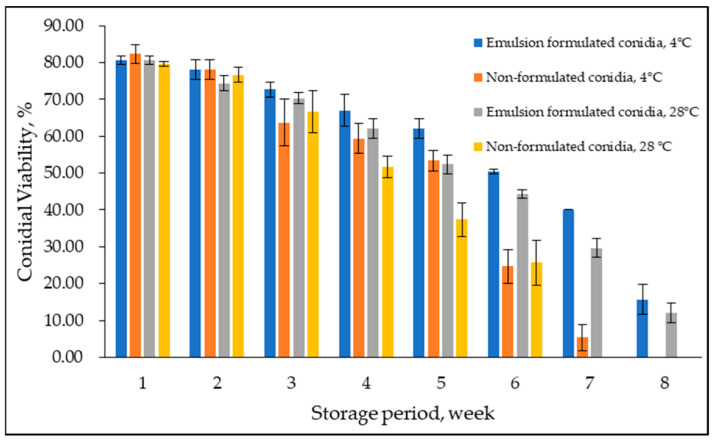
Effect of prolonged storage on emulsion formulated and non-formulated conidial viability at 4 °C and 28 °C. Bars represent mean ± standard deviation.

Conidial viability of emulsion-formulated conidia and non-formulated control was examined at 4 °C and 28 °C for eight weeks (Figure 7). Overall, conidial viability declined over time; delayed germination was observed in formulated conidia in emulsion F25. A considerable difference between treatments was observed at week six with lower conidial viability at both temperatures. In addition, in week eight, zero conidial viability was observed at 4 °C and 28 °C for non-formulated conidia in a water suspension, while formulated conidia maintained the same as 12–15% conidial viability at week eight and week nine and were observed to be not viable at week ten (not shown in the graph). Levene’s test of equality of error variances indicated sufficient evidence to claim that the variances were not equal (*F*_0.05,31,64_ = 2.999, *p* < 0.05). Additionally, the three-way ANOVA analysis showed no significant interaction between formulated and non-formulated conidia, storage period, and storage temperature (*p* = 0.108). However, the storage period significantly interacted either with emulsion-formulated and non-formulated conidia or storage temperature (*p* < 0.05).

### 3.5. Conidial Viability under Heat Stress

**Figure 8:**
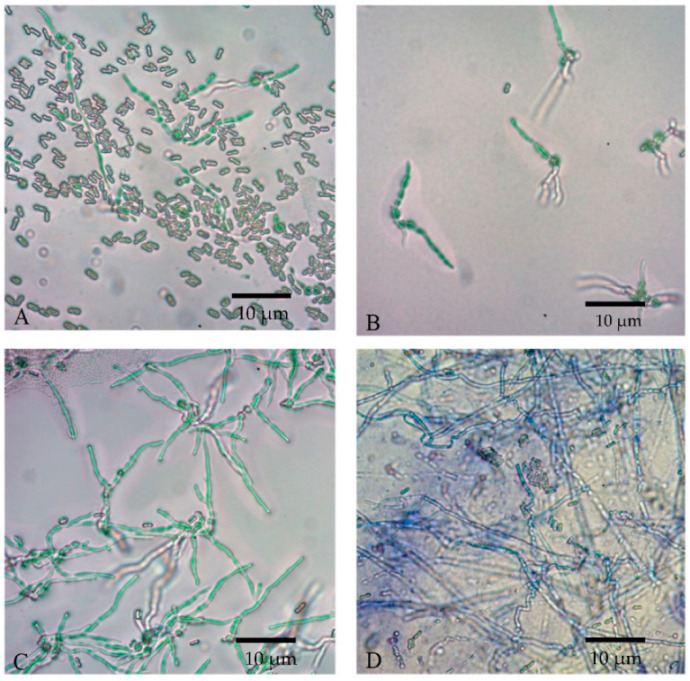
Conidial germination of *M. anisopliae* spores at 25 °C at different times; (**A**) 0 h, (**B**) 4 h, (**C**) 8 h, and (**D**) 12 h at microscopic magnification of 400×.

Conidial viability was observed at 0, 4, 8, and 12 h, as indicated by the germ tube formation, shown in Figure 8. The longer the incubation period, the denser the germinated conidia could be seen under a microscope. Both the formulated and non-formulated conidia were grown in a similar pattern.

**Figure 9:**
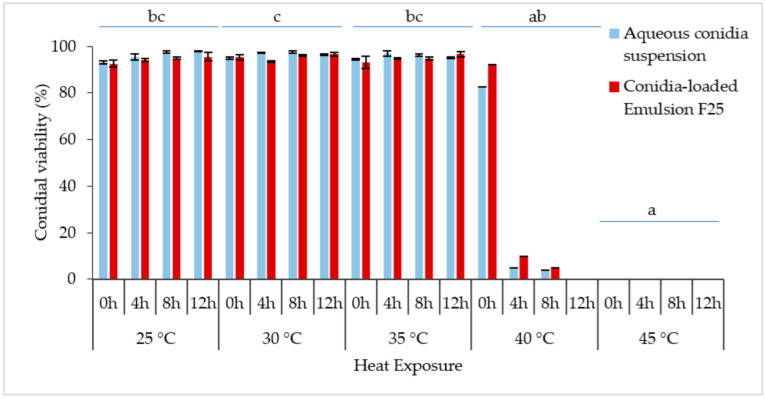
The conidial germination rate (%) of *M. anisopliae* at different temperatures (25 °C, 30 °C, 35 °C, 40 °C, and 45 °C) (*n* = 40). Bars represent mean ± standard deviation. No significant difference was detected between aqueous suspension and emulsion, thus temperature treatments followed by different letters denote significant difference at *p* < 0.05. (Kruskal–Wallis, α = 0.05); significant values were adjusted by Bonferroni correction by multiple tests.

The constant exposure to non-heat stress (25 °C) and medium heat stress (30 °C or 35 °C) did not affect the relative germination of the conidia at 0–12 h of incubation (Figure 9), reaching more than 90% conidial viability with no significant differences. Overlapping of the densely developed mycelium was observed; thus, plate incubation over 12 h was not documented. The Kruskal–Wallis H test showed that there was a significant difference in conidial germination between different temperature treatments, [χ^2^(4) = 30.198; *p* < 0.001], with a mean rank score of 27.19 for 25 °C, 31.06 for 30 °C, 27.25 for 35 °C, 11.50 for 40 °C, and 5.50 for 45 °C. 

Generally, conidial germination decreased with higher temperature and incubation period, supported by the Kruskal–Wallis tests. The increased temperature from 25 °C to 40 °C caused the germination rate to be drastically reduced from 82.58% to 3.86% within 0–8 h of the incubation period. When the incubation time reached 12 h, the germination rapidly reduced to 0%. In comparison, incubation of formulated and non-formulated conidia at 45 °C confirmed lethal for Met-Gra4 conidia. Interestingly, the formulated spores showed slightly higher conidial viability when exposed to 40 °C incubation. Pairwise comparison of sample average rank (*p* < 0.05) indicated a significant difference between conidial incubation at 45 °C and all temperatures except for 40 °C; meanwhile, there was also a significant difference between the average ranks of 40 °C and 30 °C. 

## 4. Discussion

### 4.1. Development of Emulsion Formulation

Even though fungal pathogens are potentially effective alternatives for chemical pesticides, their efficacy needs to be substantially improved at a reduced cost in order to compete with synthetic insecticides. Mycoinsecticides are practically applied at a much greater conidial dose rate than some of the latest synthetic insecticides, and they provide less hazardous effects on human safety and the environment [19]. From this study, the oil-in-glycerol emulsion was the only formulation that successfully met the criteria evaluated in the laboratory studies in terms of low-cost fabrication, emulsion stability, conidial persistence and preservation, and usability. 

Overall, the oil-in-glycerol emulsion in this study can be categorized as a nanoemulsion (NE), with its droplet sizes ranging between 150–236 nm in diameter. This oil emulsion can be defined as a kinetically stable isotropic system wherein two (or more) immiscible phases (water and oil) are blended into a single-phase mixture with appropriate surfactants [20], while its nanometric droplet sizes range between 1–200 nm. However, this is often confused with microemulsion (ME) terminology [21,22,23], as univocally several researchers did not report the various overlapped critical particle sizes, with the upper limit set at 100 nm, 200 nm, or 500 nm. NEs generally have spherical micellae since the interfacial area is reduced due to the small diameter and the high interfacial tension [23]; on the contrary, low interfacial tension makes ME droplets appear other than spherical, namely plane-like or sponge-like, depending on the type of surfactants and oil content. 

This study elucidated that the highly viscous castor oil (mostly comprised of LCTs) can only form a milky emulsion that presents a smaller isotropic region than capric oil (MCT) in the pseudo-ternary phase diagram. These non-equilibrium emulsion systems may undertake a breakdown process of coalescence, flocculation, and Ostwald ripening [24]. Several authors reported difficulty in creating nano- or microemulsions with LCTs due to the differential viscosity of the triglycerides. There is always an optimal range in any homogenization process where droplet disruption is the most efficient; Stang et al. [25] stated that the shear force in turbulent flow ηD/ηC (viscosity ratio of dispersed phase to the continuous phase) ranged between 0.1 and 1. When oil viscosity increases, droplets are poorly deformed before the flow field, causing the droplets to rotate. A study by Wooster et al. [26] found that the difference in the oil phase between hexadecane (ηD/ηC = 3.4) and a highly viscous triglyceride (ηD/ηC = 56) was the main problem in creating a triglyceride nanoemulsion due to higher oil viscosity. 

The result of using capric/caprylic triglyceride (MCT) in this study is in accordance with a previous study by Ahmed et al. [27], and similarly lower ζ-potential absolute values were reported by Taha et al. [28], wherein their MCT nanoemulsions maintained an optically isotropic system as compared to LCTs (corn oil and orange oil). In contrast, the SCT emulsions were highly unstable and susceptible to Ostwald ripening due to the relatively higher water solubility of the low molecular weight triacylglycerol [26,29]. Ostwald ripening explains the reason why larger oil particles increase at the expense of smaller ones due to the chemical potential of oil which dissolves through the aqueous phase. The motivity for micelle growth is the increased oil solubility in the aqueous phase for micellae with high curvatures [29].

Besides the influence of the oil phase, Meroni & Raikos [30] reported that the chilled storage condition had an inhibitory effect on Ostwald ripening. A four-fold mechanism can also explain this phase separation process: dissolution–ripening–regrowth–relaxation [31]; the particles tend to grow anisotropically at elevated temperatures, and high supersaturation leads to kinetic roughening of emulsion particles. However, the results showed a stable oil-in-glycerol emulsion, which coalesces at a meagre rate and is contributable to the adequate shelf life of an emulsion. Stable emulsions are formed if no phase separation is observed for a defined period and defined conditions, and if coalescence occurs, it is practically reversible.

Additionally, glycerol is suitable as a co-surfactant and in dispersed phases due to its salting-in effect, where glycerol can be incorporated into the surfactant layer, hence increasing the interfacial fluidity [32]. Many of the research studies claimed that the increasing surfactant-to-oil ratio is likely to produce a smaller droplet size [33]. However, the surfactant amount used in this study was strictly monitored and determined by building a pseudo-ternary phase diagram to obtain an optimal surfactant-to-oil ratio. Therefore, a better toxicological profile of surfactants (preferably less than 10%) toward the *M. anisopliae* conidia and the agricultural environment can be guaranteed by using this protocol. NE has several unique characteristics, such as non-toxicity, high hydrophobic active ingredient solubilization capacity, and the ability to be formed spontaneously without needing high-shear equipment, mainly due to the surfactant composition. A series of non-ionic surfactants from Span and Tween, namely Span 20, Span 80, Tween 20, and Tween 80, were selected and used in varying combinations to provide desired hydrophilic–lipophilic balance (HLB) values following the nature of the oil. 

In the present work, an oil-in-glycerol nanoemulsion was stabilized with the non-ionic surfactant mixture of Tween 20 (polyoxyethylene sorbitan monolaurate) and Span 80 (sorbitan monooleate). Both Tween and Span series of surfactants are normally recognized as safe and are approved for use in several biopharmaceutical, cosmetic, and food consumables because of their non-irritant properties and low potential for toxicity [34,35]. During the pre-formulation procedure, the HLB value or critical micelle concentration (CMC) of surfactants is crucial to be obtained; no detailed investigation was done for this study but we referred to recommendation reviews for the optimization process. Since Tween 20 has bulky hydrophilic oxyethylene (O-CH_2_-CH_2_) units with an HLB of 16.7, it tends to form oil-in-water emulsions, while Span 80 (HLB = 4.3) is a viscous lipophilic emulsifying liquid agent with a distinct hydrophobic tail, which tends to form water-in-oil emulsions. Rahate & Nagarkar [36] indicated that non-ionic surfactants are highly resistant to freezing and establish strong hydrogen bonds with water, ultimately stabilizing the emulsion. 

The ability of surfactant mixtures to enhance the long-term emulsion stability where the solubilization maximum happens for a particular proportion of the Span/Tween ratio rather than a certain HLB or surfactant hydrophobicity has been confirmed by many authors [37,38] in comparison with the use of a single surfactant. According to Stokes’ law, the smaller the diameters of oil droplets present in the emulsion, the lower their settling velocity, therefore providing more stability [39]. In this study, instability likely occurred because the non-ionic surfactants were sensitive to temperature changes. Conversely, the formulation stored at 4 °C was more stable for over one year due to the droplets’ low temperature and consequent low collision energy.

Although the natural polymers, namely wheat starch, cellulose, potato starch, and rice starch, were reported as an efficient co-stabilizer besides non-ionic surfactants [40], this unmodified native starch was incompatible with the oil-in-glycerol emulsion in this study. Less hydrophobic starch particles had considerably smaller surface cationic charge density at the starch–oil–glycerol interface. Henceforth, the Coulombic repulsion through the oil was smaller and insufficient to prevent the starch polymers from aggregating or sedimenting in the emulsion system [41]. A viscoelastic gel-like emulsion formed at the starch gelatinization temperature of 80 °C stabilized the emulsion [41]. However, it was still unsuitable for conidial formulation, wherein the conidia trapped in the starch granules may not directly adhere to the insect host. Hence, gelatinized starch is used as a growing medium for most EPF. Hitherto, only octenyl succinic anhydride (OSA) modified gelatinized starch has been recognized to adsorb at emulsion micellae interfaces, acting as emulsifiers [40], while native starches tend to flocculate with a network of micelles that appear to be spanning in congruence with the viscoelastic solid- or gel-like texture, as obtained in the current study. Overall, many crucial parts during the pre-formulation process still need to be further investigated for better emulsion stability.

### 4.2. Thermal Storability of Conidial Emulsion Formulation

The emulsion obtained in the present study mainly consisted of oil (12% *w*/*w*) and glycerol (<80% *w*/*w*), which can prevent viable conidia’s premature germination. As calculated by Lazzarini et al. [42], the high water content may induce germ tube formation within the emulsion system (with a water activity threshold of 0.94 a_w_ or 95.5% relative humidity), while 0.93 a_w_ may be unfavorable for conidial germination, but it is a fungal strain-dependent condition. Therefore, germ tube formation of *M. anisopliae* should be activated upon dilution of an oil-in-glycerol stock emulsion to optimize water activity before field application. Since the EPF conidia actively attach to the host cuticle through surface hydrophobic protein interaction followed by hyphal penetration and growth on nutrient-rich hemocoel [43], the EPF may then employ numerous secondary metabolites to overcome the host response. After host death, EPF changes to saprophytic growth. With over 95% relative humidity, conidiation occurs across insect cuticles. Inversely, under low relative humidity, the mycelium might proliferate internally, developing chlamydospores. Thus, pre-germinated conidia may be deficient in nutrients and become non-viable inside a stock emulsion, referring to the oil-in-water system [44].

In terms of the shelf life of this industrially important EPF, glycerol in the emulsion F25 acts as a microbial cryoprotectant for cold storage while prolonging emulsion stability. A study by Ryan et al. [45] elucidated the inclusion of glycerol for *M. anisopliae* and *Fusarium oxysporum* metabolic integrity to less than one year in water under −20 °C; however, the stability of secondary metabolite profiles deteriorated over more extended storage periods. The findings by Patel et al. [32] also supported that glycerol stabilizes the native structure of globular proteins and protects cells upon drying and freezing and suggested the drawback of continual fungal plate culture compared to cryopreservation. 

Additionally, high-glycerol conidia of non-osmophiles, such as *M. anisopliae* and *Paecilomyces farinosus*, were able to form germ tubes at reduced water activities (≤0.89 a_w_) than low-glycerol spores at more than 0.99 a_w_ [46], wherein glycerol serves in scaling up the water activity limit for fungal cell function, concerning spatiotemporal constraints of the fungal habitat. A liquid bioformulation of *M. anisopliae* developed by Boruah et al. [47] using different adjuvants and oils was also reported to facilitate fungal growth when blended with glycerol (10%) and sunflower oil (0.5%) while enhancing insect mortality up to 80% against cowpea aphids. The present study also demonstrated that the oil-induced enhancement of insect mortality is in agreement with several reports [48,49]. Despite the total loss of conidial viability after 10 weeks of storage, the decrease in viability is still acceptable for practical use or in the development of emulsion formulation for crop protection purposes. 

Based on the loss of *M. anisopliae* conidia viability at 35 °C, the data indicated increasing conidial death due to prolonged heat stress exposure. Varying conidial moisture content may account for the variations observed between the treatments. Nonetheless, the oil-in-glycerol formulation could not provide adequate thermal protection to the non-formulated conidia at 40 °C. The protective suspending oil–glycerol mixture could have prevented conidial debilitation by lethal imbibitional damage through fast and direct rehydration upon dilution [50]. In fact, during field application, one should not presume growers to gradually rehydrate dry-harvested conidia, especially in a large-scale application when a considerable number of fungal propagules is involved. Depending on *M. anisopliae* isolate, Xavier-Santos et al. [44] claimed that water with a temperature below 45 °C caused no inhibitory effect on dry conidia but prolonged immersion time was able to debilitate or slow down germination, reducing conidial infectivity. 

Unfavorable conditions for fungal disease incitation and survival partially elucidate the low quality and inconsistent results in several studies. Ultraviolet irradiation, relative humidity, and temperature are the crucial ecophysiological factors influencing the survival and infectivity of EPF [51]. Therefore, vast arrays of studies have examined abiotic effects on the biological parameters of the EPF, namely *B. bassiana* and *M. anisopliae,* in vitro. However, it is debatable whether laboratory results can be extrapolated to field conditions [52] because of environmental factors on fungal performance such as infection potential, conidial persistence, and complex host–fungi ecological interactions, infrequently duplicated in laboratory environments.

Survival of EPF under unconducive environments could be vital for their success as a biocontrol agent, and understanding the survival mechanism of EPF in myriads of host environments is essential to developing mycoinsecticides. The present study elucidated that *M. anisopliae* strain Met-Gra4 with its infective conidia was hypervirulent against adult weevil, *R. ferrugineus*, particularly the population found in Terengganu, Malaysia as compared to the study of non-formulated conidia by Grace et al. [15]. The percentage of conidial viability in this study showed a general pattern of declined conidial viability over time, regardless of storage temperature. However, conidia formulated in the oil-in-glycerol system positively retained a higher percentage of viable conidia at optimal and low ambient temperatures. Environmental conditions close to EPF survival limits can drive local adaptation when these limits are constantly encountered [53]. The optimal temperature for growth and entomopathogenicity against insect hosts of *Metarhizium* spp. and *Beauveria* spp. is usually between 25 °C and 30 °C [54,55]. However, a discrepancy exists in the EPF thermal preference and field efficacy on target hosts [53], and each fungal strain of the same species can differ in its thermal optima [56].

## 5. Conclusions

In conclusion, most commercialized vegetable oils can create a stable micro- or nano-emulsion with the aid of non-ionic surfactants. Several combinations of inert ingredients were evaluated and an emulsion system named F25 was selected based on the emulsion stability at room temperature. Emulsion F25 achieved stable oil-in-glycerol mixtures with 0.1–10% of the active ingredient (*M. anisopliae* conidia), recorded at the ratio of 12% capric oil, 8% Tween 20/Span 80 surfactant blend (1:1), and 80% aqueous glycerol. The one-phase transparent system indicated a stable nanoscale oil and water molecules dispersed in glycerol with the aid of the Tween 20/Span 80 surfactant blend. Overall, the vegetable oils served as conidial carriers rather than providing deleterious effects on the conidial viability and can proceed with further thermal tolerance and pathogenicity assessment.

Besides that, even though the conidial viability declined over time; delayed germination was observed in emulsion-formulated conidia. A low temperature of 4 °C was suggested to be the best storage temperature for extended shelf life, up to eight weeks. Overall, the shelf life of viable conidia in the emulsion F25 was prolonged in comparison with non-formulated conidia. Surprisingly, there was not much difference in thermal protection for conidia in the oil-in-glycerol emulsion, as reported by several studies. The oil-in-glycerol formulation did not provide sufficient thermal protection to the non-formulated conidia at 40 °C. However, the protective suspending oil–glycerol mixture prevented conidial debilitation by lethal imbibitional damage through fast and direct rehydration upon dilution. The prolonged exposure to temperatures above 35 °C in the present study simulated extreme conditions as encountered by growers during insecticide preparations and the interval period before spray application.

## Figures and Tables

**Table 1:** Combination of ingredients tested for emulsion system.

Oil	Emulsifier (% *w*/*w*)	Span 80: Tween 20	Span 80: Tween 80	HLB Number of Surfactant Blend	Required HLB of Oil
Canola oil	<20%	8:2	7:3	6.78–7.51	7
Capric oil	<20%	1:1	1:1	9.65–10.5	11
Sunflower oil	<20%	8:2	7:3	6.78–7.51	7
Sesame oil	<20%	8:2	7:3	6.78–7.51	7
Castor oil	<20%	1:9	2:8	12.86–15.46	14

**Table 2:** Effect of storage duration on the characteristics of emulsion F25 at room temperature. Data expressed as mean ± standard deviation (*n* = 3).

Emulsion F25	Storage Duration (Days)	Z-Average (d.nm ± SD)	Polydispersity Index (pdi ± sd)	Zeta Potential (mv ± sd)	Surface Tension (mN/m ± SD)
Blank	0	154.3 ± 1.4	0.257 ± 0.012	36.6 ± 0.6	29.83 ± 0.24
	30	213.2 ± 4.5	0.297 ± 0.000	36.6 ± 0.5	
	60	162.2 ± 3.4	0.272 ± 0.000	36.8 ± 1.2	
	90	236.4 ± 1.3	0.281 ± 0.017	36.2 ± 1.4	
Conidia-loaded	0	220.9 ± 2.2	0.348 ± 0.000	37.2 ± 0.8	30.72 ± 0.11
	30	805.2 ± 135.4	0.873 ± 0.016	15.3 ± 1.7	
	60	804.5 ± 49.4	0.759 ± 0.002	16.0 ± 0.7	
	90	803.3 ± 48.4	0.800 ± 0.040	15.5 ± 0.6	

## Data Availability

Not applicable.
